# Etirinotecan pegol administration is associated with lower incidences of neutropenia compared to irinotecan administration

**DOI:** 10.1007/s00280-016-3192-6

**Published:** 2016-11-30

**Authors:** S. Kenneth Sy, Theresa D. Sweeney, Chunmei Ji, Ute Hoch, Michael A. Eldon

**Affiliations:** 1 Department of Clinical Pharmacology, Nektar Therapeutics, 455 Mission Bay Boulevard South, San Francisco, CA 94158 USA; 2 Department of Toxicology, Nektar Therapeutics, San Francisco, CA USA

**Keywords:** Etirinotecan pegol, Irinotecan, SN38, Neutropenia, Breast cancer, NKTR-102

## Abstract

**Purpose:**

The relationship between incidences of neutropenia and 10-hydroxy-7-ethyl camptothecin (SN38) exposure was explored using SN38 pharmacokinetic and neutrophil count data from toxicology studies of etirinotecan pegol (EP) and irinotecan in beagle dogs.

**Methods:**

Dogs received four weekly intravenous infusions of either vehicle control (*n* = 22), EP (6, 15, 20, 25, 40/25 mg/kg; *n* = 3–9 dogs/dose group/sex; *n* = 48), or irinotecan (20 or 25 mg/kg *n* = 3–4 dogs/dose group/sex; *n* = 14). Blood samples were collected up to 50 days post-dose for characterization of SN38 pharmacokinetics. Two separate models were created describing SN38 concentration time profiles after either irinotecan or EP administrations to project the AUC_0–168h_ after Day 1 and Day 22 doses. The relationship between incidence of neutropenia and SN38 exposure was explored using logistic regression.

**Results:**

The incidence of neutropenia in dogs receiving weekly doses of irinotecan or EP was strongly correlated with maximum plasma SN38 concentration (*C*_max_), but not SN38 area under the concentration–time curve (AUC). Neutropenia occurred in approximately 80% of dogs receiving irinotecan (mean SN38 *C*_max_ of 13.5 and 26.3 ng/mL for 20 and 25 mg/kg, respectively). No neutropenia occurred in dogs receiving EP at doses up to and including 25 mg/kg (mean SN38 *C*_max_ of 3.4 and 4.9 ng/mL for 20 and 25 mg/kg, respectively), despite 2.5–3.6 times greater SN38 AUC after EP compared to irinotecan at equivalent doses.

**Conclusions:**

EP administration avoids both high SN38 *C*_max_ values and development of dose-limiting neutropenia observed after irinotecan, while maintaining greater and sustained SN38 exposure between doses.

**Electronic supplementary material:**

The online version of this article (doi:10.1007/s00280-016-3192-6) contains supplementary material, which is available to authorized users.

## Introduction

Irinotecan is the active pharmaceutical ingredient of Camptosar^®^ (Camptothecin-11), a topoisomerase 1 inhibitor widely used as a chemotherapeutic agent. Irinotecan is metabolized via enzymatic cleavage of the C-10 side chain by carboxylesterases to generate the biologically active metabolite, SN38, which is 100- to 1000-fold more potent as a cytotoxic agent than irinotecan [1]. Although irinotecan has clinical utility, its anti-tumor activity may be limited by its short half-life due to inactivation at physiological pH by the opening of its lactone E-ring and rapid clearance of both parent drug and SN38. In humans, the terminal half-life (*t*_1/2_) of irinotecan is 9–14 h, while the *t*_1/2_ of SN38 is 24–47 h [2–4]. The recommended irinotecan dosing regimen of 350 mg/m^2^ administered as a 90-min infusion every 21 days results in high SN38 *C*_max_ near the end of infusion. SN38 exposure was previously shown to be a strong determinant of neutropenia [5–8]. The side effect profile of irinotecan is dependent on the mode of administration, with protracted infusions associated with lower incidences of severe myelosuppression [7–9], suggesting that SN38 *C*_max_ rather than duration of exposure contributes to severe hematologic toxicities.

Because SN38 inhibits DNA topoisomerase I during the S-phase of the mitotic cycle, it is believed that extended SN38 cytotoxic exposure would enhance anti-tumor activity [10, 11]. Additionally, the conversion of irinotecan to SN38 by carboxyl-esterase enzymes is believed to be a saturable process [9]. In patients with metastatic colorectal cancer, continuous infusion of 22.5 mg/m^2^/day given over 7 days, every 21 days, for a total of 157 mg/m^2^ per 21-day cycle, resulted in greater SN38 AUC compared to the 350 mg/m^2^ given an 90-min infusion, even though only half the dose was administered [7]. A study investigating continuous infusion of irinotecan over 14 days in patients with malignant solid tumors also reported a greater SN38 AUC compared to short infusion of equivalent irinotecan dose [8].

EP, a long-acting topoisomerase 1 inhibitor designed to provide sustained exposure to SN38, was developed with the aim of providing increased anti-tumor activity and improved safety compared with short-acting topoisomerase 1 inhibitors [12]. EP is composed of irinotecan conjugated with polyethylene glycol via an ester-based linker that slowly releases irinotecan, a prodrug of SN38. The in vivo hydrolysis of this cleavable linker results in slow formation of irinotecan and subsequently slower transformation to SN38, resulting in reduced *C*_max_ yet sustained plasma SN38 concentrations.

We hypothesized that the pharmacokinetic properties of EP would attenuate *C*_max_, sustain exposure, and result in higher AUC to the active metabolite SN38 without encountering hematologic toxicities compared to irinotecan. To test this hypothesis, the relationship between incidences of neutropenia and model-predicted SN38 exposure was evaluated using SN38 *C*_max_ and AUC and neutrophil count data from toxicology studies in beagle dogs receiving EP or irinotecan once weekly for 4 weeks.

## Materials and methods

### Chemicals and reagents

EP (Nektar Therapeutics, San Francisco, CA) was dissolved in 5% dextrose in water adjusted to a final pH of 5.20–5.86. To facilitate direct comparison with irinotecan, all EP doses are expressed as irinotecan-equivalent unit delivered in the conjugated form. Irinotecan (Camptosar^®^, Pfizer, Groton, CT) was also prepared by dissolution in 5% dextrose in water, and final pH was adjusted to 4.61–4.85. The control solution contained only the vehicle, which was 5% dextrose in water.

### Animals

Eighty-four beagle dogs equally divided by gender were supplied by Marshall Bioresources (North Rose, NY). The age range of the dogs was 6–7 months old at the onset of experiment. The protocol was reviewed and approved by the Animal Care Committee of ITR Laboratories Canada, Inc. where the study was conducted. All animals were handled in accordance with the principles outlined in the Guide to the Care and Use of Experimental Animals [13, 14] and Guide for the Care and Use of Laboratory Animals [15].

### Dog toxicokinetics

All animals were monitored for clinical condition, body weight, food consumption, and mortality throughout the study. Ophthalmological and electrocardiographic evaluations were performed. Blood samples were collected for evaluation of hematology and toxicokinetics.

The experimental design is shown in Table 1. Beagle dogs (42 males and 42 females) were assigned to vehicle control (*n* = 22), 20 (*n* = 6), or 25 (*n* = 8) mg/kg doses of irinotecan or 6 (*n* = 10), 15 (*n* = 10), 20 (*n* = 6), 25 (*n* = 8), or 40/25 (*n* = 14) mg/kg doses of EP. Each dose group was further divided into main (3 of each gender), 14-day recovery (2 of each gender), and 28-day recovery groups (2 of each gender only in the control and high-dose groups). The study phases were defined by the time that the dogs were euthanized post-first dose, either Day 25 (main study), Day 37 (14-day recovery), or Day 51 (28-day recovery).

**Table 1:** Experimental design showing the assignment of number of animals to each study group, the dose received, and the phases of the study

Group no.	Test article	Dose level (mg/kg/week)^a^	Dose conc. (mg/mL)^b^	Main (Day 25 Nec)		Recovery phase			
						14 days (Day 37 Nec)		28 Days (Day 51 Nec)	
				M	F	M	F	M	F
1	Vehicle	0	0	6	6	3	3	2	2
2	EP	6	0.75	3	3	2	2	nd	nd
3	EP	15	1.875	3	3	2	2	nd	nd
4	EP	20	2.5	3	3	nd	nd	nd	nd
5	EP	25	3.125	3	3	1	1	nd	nd
6	EP	40/25	5/3.125	3	3	2	2	2	2
7	Irinotecan	20	2.5	3	3	nd	nd	nd	nd
8	Irinotecan	25	3.125	3	3	1	1	nd	nd

*EP* etirinotecan pegol, *M* male, *F* female, *Nec* necropsy

^a^In mg irinotecan contained

^b^Animals in the high-dose (40/25) etirinotecan pegol group were dosed at 40 mg/kg for the first two treatments (Days 1 and 8) and were then dosed at 25 mg/kg on Days 15 and 22 due to severe adverse clinical signs and mortalities observed on Day 8. However, for two animals in the first replicate, the dose level was decreased to 25 mg/kg only for Day 22, since they were dosed on Day 15 prior to the decision to change the dose level

The control/vehicle and test article solutions (dose volume 8 mL/kg) were administered to each dog by intravenous infusion over a 1-h period via an indwelling catheter placed in the cephalic or saphenous vein on Days 1, 8, 15, and 22. In the EP 40/25-mg/kg dose group, animals were administered 40 mg/kg on Days 1 and 8 followed by 25 mg/kg on Days 15 and 22 due to severe adverse clinical signs and mortalities observed on Day 8. For the first two dogs evaluated in the 40-mg/kg dose group, the dose level was decreased to 25 mg/kg on Day 22, as they had already received their Day 15 dose before the decision to change the dose level was made. Two male animals in the EP 40/25-mg/kg dose group only received three (Days 1, 8, and 22) of the four scheduled doses due to poor health. In the irinotecan-treated animals, two animals in the 20 mg and 25-mg dose groups received humane euthanasia on Days 6 and 13, respectively. Three animals in the 25-mg dose group were found dead on Days 5 (*n* = 2) and 21 (*n* = 1).

Blood samples were collected for toxicokinetic evaluation in the main study group: pre-dose on Days 1, 15, 22 and post-dose at 1, 8, 48, 96, and 168 h on Day 1, and at 1, 8, and 48 h on Day 22. An additional morning sample was collected on Day 36 for the 14-day recovery group, while the 28-day recovery group had additional samples taken on the morning of Days 38 and 50. Blood samples were analyzed for plasma concentrations of EP, irinotecan, and SN38, as well as hematologic parameters.

### Pharmacokinetic analysis

Plasma concentrations of SN38 were quantified by a validated liquid chromatography–tandem mass spectrometry method as previously described [12].

The observed *C*_max_ was determined directly from the concentration–time profiles. The area under the plasma concentration–time curve from time 0 to *t* (AUC_0–*t*_), wherein *t* is defined as either 48 or 168 h, was calculated using both linear trapezoidal method and a nonlinear mixed effect modeling approach. All concentrations reported as below the limit of quantitation were set to missing in the computation.

### Pharmacokinetic model description

To obtain comparable SN38 AUC values for all dogs after irinotecan and EP administrations, simulated concentration–time profiles based on nonlinear mixed effects modeling were used. Determination of *C*_max_ relied on the experimentally observed values. The area under the plasma concentration–time curves from time 0 to *t* (AUC_0–*t*_), wherein *t* is defined as either 48 or 168 h, were calculated by integration using the developed model as described below.

SN38 pharmacokinetics in dogs after EP administration was characterized by a two-compartment model with first-order input describing the slow increase in plasma SN38 concentration. The structural model relative to EP dose was parameterized on clearance (CL/*F*), intercompartmental clearance (*Q*/*F*), volumes of central (*V*_c_/*F*) and peripheral (*V*_p_/*F*) compartments, and the rate constant that described the appearance of SN38 in the plasma (*K*_in_). Between-subject variability (BSV) was characterized by an exponential model on CL/*F*, *V*_c_/*F*, and *Q*/*F*, whereas between-occasion variability (BOV) was associated with CL/*F* and *V*_c_/*F*, also as an exponential model, such that:

$$P_{ij} = \bar{P} \times \exp \left( {\eta_{i} + \kappa_{ij} } \right)$$

where $\bar{P}$ represents the mean parameter value. *η*_*i*_ and *κ*_*ij*_ describe the BSV and BOV, both of which were assumed to be normally distributed with mean zero and variances of *ω*_*p*_^2^ and *π*_*p*_^2^, respectively. The subscript *i* represents the individual, and *j* represents the occasion for the individual. Dummy variables were introduced to distinguish separate occasions, where 1 designated the first occasion for all observation times prior to the fourth weekly dose and 2 indicated observation times after the fourth weekly dose. The two occasions were consolidated to determine the BOV.

SN38 concentration–time profiles in dogs after irinotecan administration were characterized by a two-compartment model with zero-order input (1-h infusion) to describe the rapid increase in SN38 concentration in plasma after 1-h infusion of irinotecan. The structural model relative to irinotecan dose was parameterized on CL/*F*, *Q*/*F*, *V*_c_/*F*, and *V*_p_/*F*. The BSV was characterized by an exponential model for all parameters except *V*_p_/*F*. There was no BOV in the model for SN38 pharmacokinetics after irinotecan administration.

The residual variability was modeled using a proportional error model for both models of SN38.

Pharmacokinetic model evaluation was based on the likelihood objective function value (OFV), goodness-of-fit plots, precision of parameter estimates, shrinkage for BSV and BOV terms and for the residual variability, bootstrap resampling, and visual predictive check [16]. Corrections for prediction and variability were incorporated in the visual predictive check [17].

AUC was calculated by integration of a separate compartment defined as the amount in the central compartment divided by *V*_c_/*F* [18]. For example, AUC_0–168h_ after the last dose was computed as total AUC_0–672h_ minus total AUC_0–504h_, and AUC_0–48h_ after Day 22 dose was computed as AUC_0–552h_ minus AUC_0–504h_. The SN38 AUC_0–48h_ values on Day 1 and Day 22 determined from non-compartmental analysis were compared to the AUC_0–48h_ values determined from the population pharmacokinetic model by correlation.

### Statistical analysis

Logistic regression analysis was performed to determine the relationship between the occurrence of neutropenia and the SN38 exposure parameters *C*_max_ and AUC_0–168h_. Observations from 82 dogs were included in the logistic regression analysis. The logit function was fitted to both *C*_max_ and AUC_0–168_ values to relate the probability of developing neutropenia:

$$P_{{\left( {Y_{i} = 1} \right)}} = \frac{1}{{1 + \exp \left( { - f\left( {\text{parameter}} \right)} \right)}}$$

where parameter refers to *C*_max_ or AUC_0–168_ values. The logit transformation limits the probability of developing neutropenia between 0 and 1. *f*(parameter) is a linear function of either SN38 *C*_max_ or SN38 AUC_0–168_:

$$f\left( {\text{parameter}} \right) = \, \propto + \beta \left( {\text{parameter}} \right)$$

where *α* and *β* represent intercept and slope of the regression. The odds ratio was defined as the change in the estimated odds of having neutropenia when *C*_max_ or AUC is increased by one unit.

The predictability of the final logistic regression model was evaluated using the visual predictive check, wherein plasma concentration–time profiles after a single 25 mg dose were simulated in 1000 replicates using the final model. The percentage of dogs with neutropenia within a specific *C*_max_ range was estimated and compared with the actual observed percentage to evaluate the predictive performance of the final model.

### Software

Nonlinear mixed effects modeling of SN38 pharmacokinetics was carried out in NONMEM (ICON, Ellicott City, MD), with first-order conditional estimation and η interaction. The subroutine was ADVAN6 with tolerance value of 9. Visual predictive check and bootstrap resampling were performed with Perl-speaks-NONMEM 3.5.5 running ActivePerl 5.12 (ActiveState, Vancouver, Canada). The non-compartmental analysis was carried out using Phoenix WinNonlin 6.3 (Certara, Princeton, NJ). The logistic regression models were developed using R (version 3.0.0).

## Results

### Comparison of SN38 *C*_max_ and AUC values after administration of irinotecan or etirinotecan pegol

**Fig. 1:**
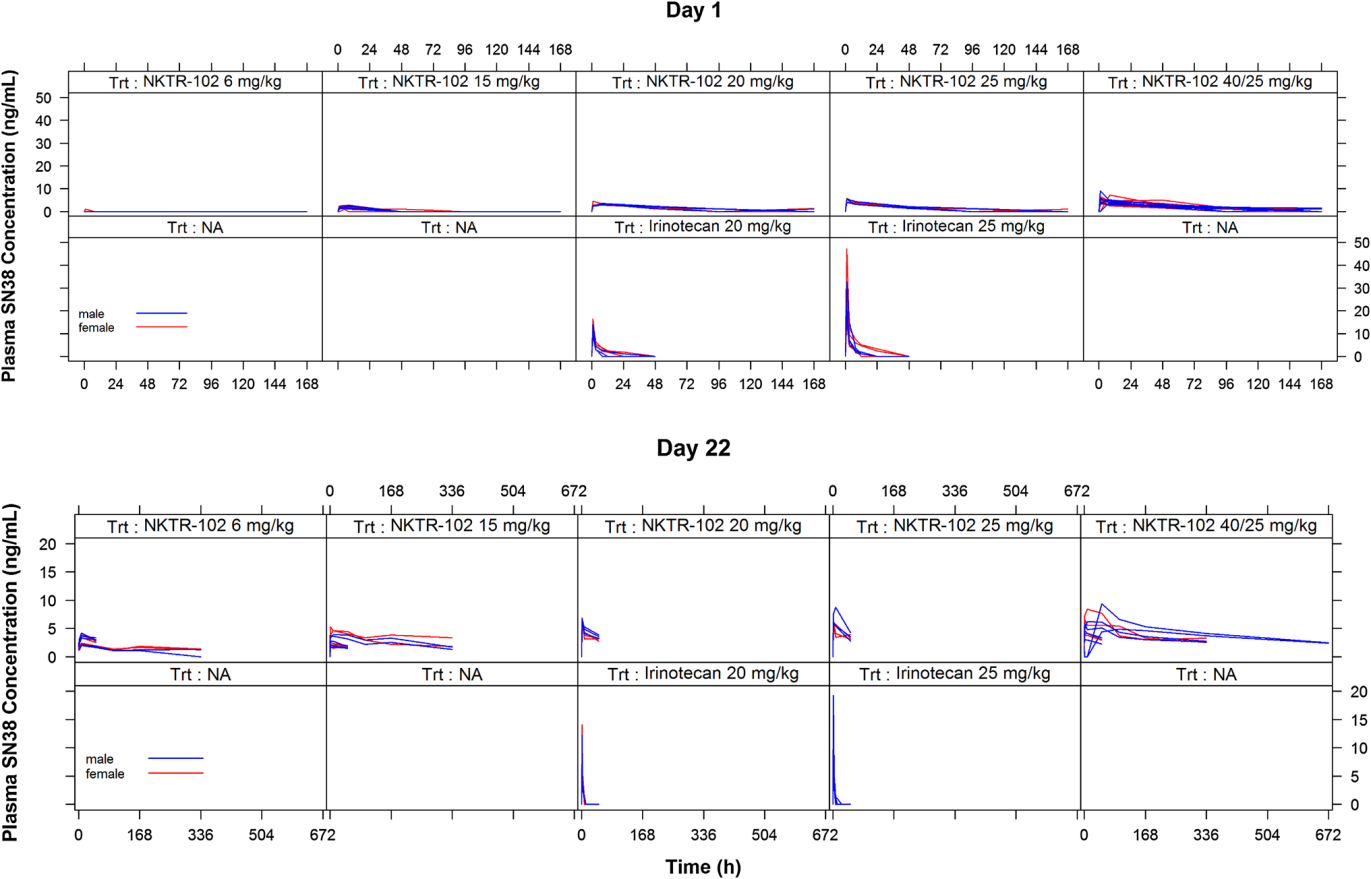
Plasma SN38 concentration–time profiles in dogs by dose group. Dogs for each treatment group were split equally between genders. The high-dose (40/25) etirinotecan pegol animals were treated at 40 mg/kg for the first two treatments (Days 1 and 8) and were then dosed at 25 mg/kg on Days 15 and 22 due to severe adverse clinical signs and mortalities observed on Day 8. However, for two animals in the first replicate, the dose level was decreased to 25 mg/kg only for Day 22, since they were dosed on Day 15 prior to the decision to change the dose level

Figure 1 shows plasma SN38 concentration–time profiles after Day 1 and Day 22 administrations by increasing EP and irinotecan doses and by gender of beagle dogs. SN38 disposition was similar between male and female dogs. The plasma disposition of SN38 after irinotecan administration is characterized by rapid increase followed by a fast exponential decline, whereas SN38 disposition after EP administration is characterized by lower *C*_max_ values, much slower elimination, and higher AUC values for irinotecan-equivalent doses. SN38 concentrations after irinotecan administration were below the limit of quantitation after 48 h post-dose, whereas SN38 concentration after EP remained measurable for the duration of assessment (48–678 h). The SN38 pharmacokinetic model development results are found in supplemental material.

**Fig. 2:**
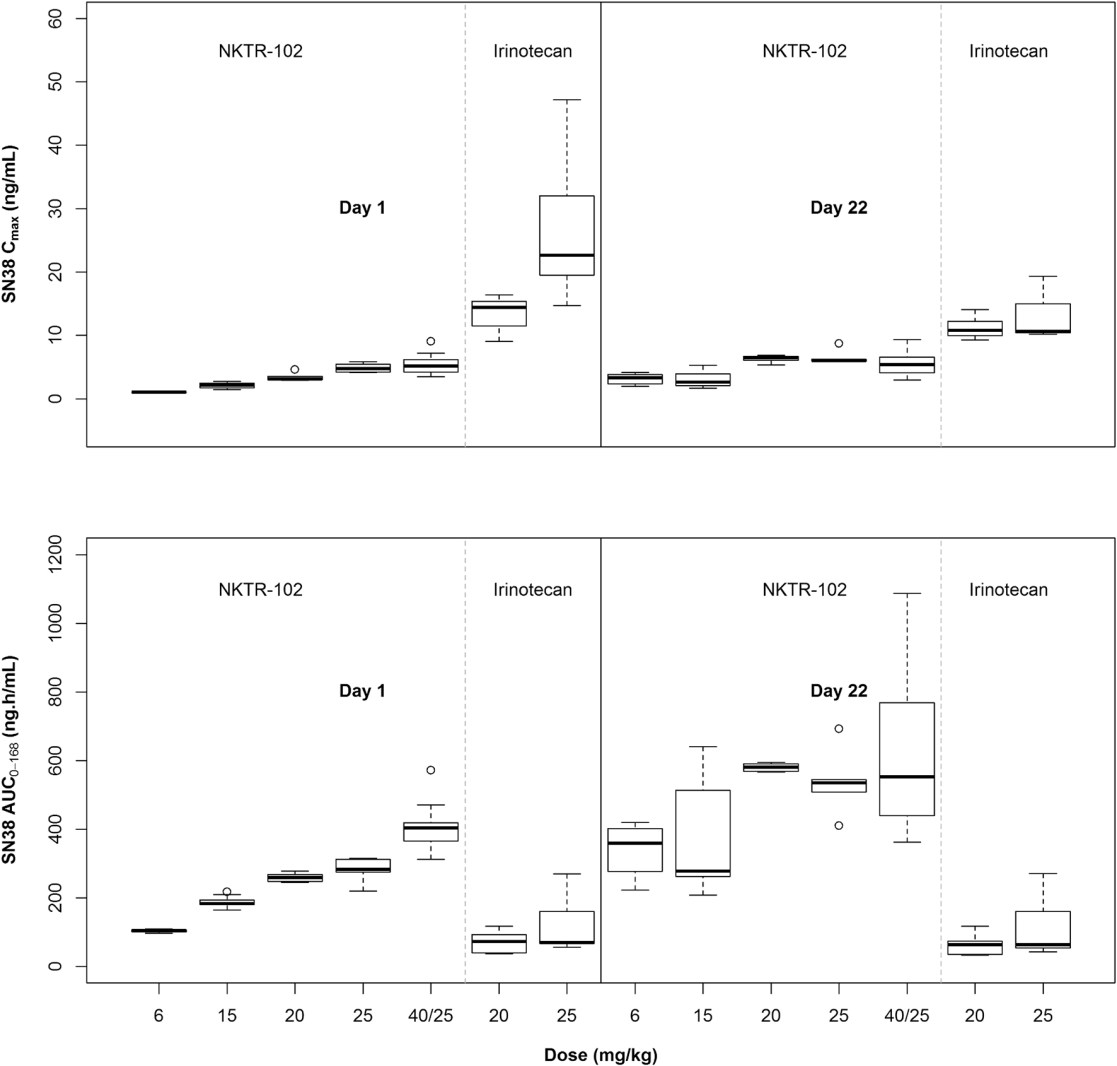
Comparison of SN38 *C*_max_ (*top*) and AUC_0-168h_ (*bottom*), by study day, treatment, and dose level

Figure 2 shows SN38 *C*_max_ and AUC after administration of irinotecan or EP; exposure parameters are summarized in Table 2. At dose levels delivering the same irinotecan content, mean SN38 *C*_max_ values after the first dose of irinotecan were approximately 4- to 5.4-fold higher than after the first dose of EP. In contrast, Day 1 mean SN38 AUC after EP was 2.5- to 3.6-fold greater than following irinotecan administration. This ratio increased 4.9- to 8.9-fold after repeated administration on Day 22. No accumulation of SN38 was observed after irinotecan dosing.

**Table 2:** SN38 pharmacokinetic parameters after 1-h intravenous infusion of irinotecan or etirinotecan pegol

Treatment	Dose (mg/kg)	*C*_max_ (ng/mL)		AUC_0–168h_ (ng h/mL)^a^	
		Day 1	Day 22	Day 1	Day 22
Irinotecan	20 (*N* = 6)	13.5 ± 2.7 [9.07, 16.4]	11.3 ± 1.9 [9.31, 14.1]	72.4 ± 31.1 [37.6, 118]	65.0 ± 30.8 [34.0, 118]
	25 (*N* = 8)	26.3 ± 10.5 [14.7, 47.2]	12.7 ± 4.4 [10.2, 19.3]	115 ± 77.0 [56.2, 270]	109 ± 82.4 [43.3, 272]
Etirinotecan Pegol	6 (*N* = 6)	1.03 [0, 1.03]	3.11 ± 0.84 [1.98, 4.18]	104 ± 3.77 [97.4, 110]	338 ± 73.3 [223, 420]
	15 (*N* = 10)	2.08 ± 0.47 [1.43, 2.74]	3.07 ± 1.25 [1.69, 5.31]	188 ± 16.4 [165, 218]	364 ± 152 [209, 640]
	20 (*N* = 6)	3.39 ± 0.63 [2.90, 4.61]	6.32 ± 0.6 [5.32, 6.84]	260 ± 13.0 [245, 279]	581 ± 12.0 [566, 595]
	25 (*N* = 6)	4.86 ± 0.75 [4.14, 5.87]	6.45 ± 1.13 [5.82, 8.74]	282 ± 34.7 [220, 316]	537 ± 90.7 [410, 693]
	40/25 (*N* = 14)	5.40 ± 1.49 [3.49, 9.08]	5.69 ± 2.01 [2.98, 9.34]	409 ± 62.7 [313, 572]	614 ± 212 [363, 1087]

Values reported as mean ± SD [min, max]

^a^AUC_0–168h_ was determined from the population pharmacokinetic model

### Relationship between decrease in neutrophils and SN38 exposure

Baseline neutrophil counts for all dogs ranged between 2.68 and 14.41 × 10^9^ cells/L. Neutropenia was defined as a decrease in neutrophil count below 2 × 10^9^ cells/L at any time during the study.

Table 3 shows the incidence of neutropenia by treatment and dose group. In the irinotecan treatment groups, 100% of dogs receiving 20 mg/kg and 60% of dogs receiving 25 mg/kg experienced neutropenia. The remaining 40% of dogs in the irinotecan 25 mg/kg group died before study completion with reduced neutrophil counts, but prior to meeting criteria for neutropenia. In the EP treatment groups, none of the dogs receiving ≤25 mg/kg developed neutropenia, but 71% of dogs receiving 40/25 mg/kg did.

**Table 3:** Incidences of neutropenia in dogs by treatment and dose groups

Treatment	Dose (mg/kg)	Number of dogs	Neutropenic dogs^a^	
		*n*	*n*	%
Control (*n* = 22)	0	22	0	0
Etirinotecan pegol (*n* = 46)	6–25	32	0	0
	40/25	14	10	71
Irinotecan (*n* = 14)	20	6	6	100
	25	8	5	60

^a^Neutropenic dogs are defined as dogs whose neutrophil count was < 2x10^9^/L at any point during the treatment

**Fig. 3:**
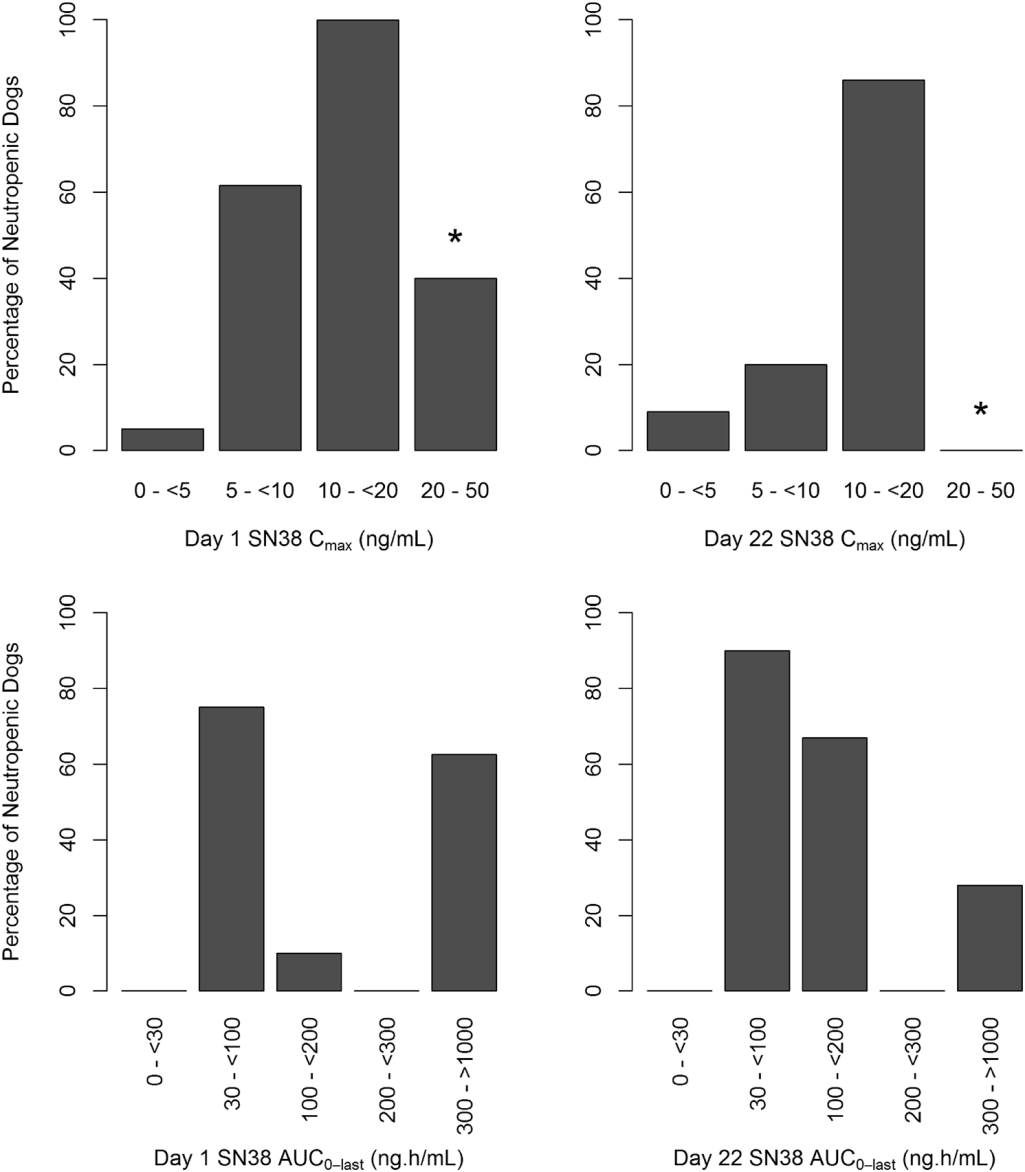
Observed fraction of dogs with neutropenia by SN38 *C*_max_ (*top*) and AUC_0–168h_ (*bottom*). Percentages are computed based on number of dogs within the bin range. *In the 20–50 ng/mL *C*_max_ range, the dogs that were given 25 mg/kg irinotecan and died prior to their neutrophil levels falling below 2 × 10^9^/L were not counted as neutropenic. Majority of the dogs in 25 mg/kg irinotecan died prior to their Day 22 dose

Figure 3 shows the incidences of neutropenia by SN38 *C*_max_ and SN38 AUC_0-168h_. The top graphs show the percentage of neutropenic dogs for binned SN38 *C*_max_ values after the first (Day 1) and fourth (Day 22) weekly dose of either irinotecan or EP. An increasing incidence of neutropenia was observed with increasing SN38 *C*_max_. SN38 *C*_max_ values <5 ng/mL were associated with low incidences of neutropenia (<10%). Incidence of neutropenia increased to 67% at SN38 *C*_max_ values between 10 and <20 ng/mL and reached 100% for SN38 *C*_max_ values between 20 and 50 ng/mL. While SN38 *C*_max_ values after administration of EP were <10 ng/mL (with 74% <5 ng/mL), irinotecan administration led to SN38 *C*_max_ values between 10 and 50 ng/mL. At irinotecan-equivalent dose levels (20 and 25 mg/kg), mean SN38 *C*_max_ values after EP administration were twofold to fivefold lower compared to irinotecan administration and did not exceed 5 ng/mL after Day 1 and 10 ng/mL after Day 22 EP administrations (Table 2).

Table 4 shows the parameter estimates for the linear logistic regression model. The Day 1 odds ratio was equal to *e* ^0.12^, corresponding to a value of 1.13, while the Day 22 odds ratio was *e* ^0.33^ or 1.39. The odds ratios for both days were statistically greater than 1, indicating that the probability of neutropenia occurring was dependent on SN38 *C*_max_.

**Table 4:** Summary pharmacodynamic parameters of SN38 *C*_max_ and neutropenia incidence relationship

Study day	Model parameters	Estimate (SE)
Day 1	*α* (logit intercept)	−1.88 (0.37)
	*β* (logit slope)	0.12 (0.038)*
	Odds ratio^a^	1.13
Day 22	*Α*	−3.43 (0.714)
	*Β*	0.33 (0.093)*
	Odds ratio^‡^	1.39

* *p* < 0.01

^a^Odds ratio is computed as exp(*β*)

**Fig. 4:**
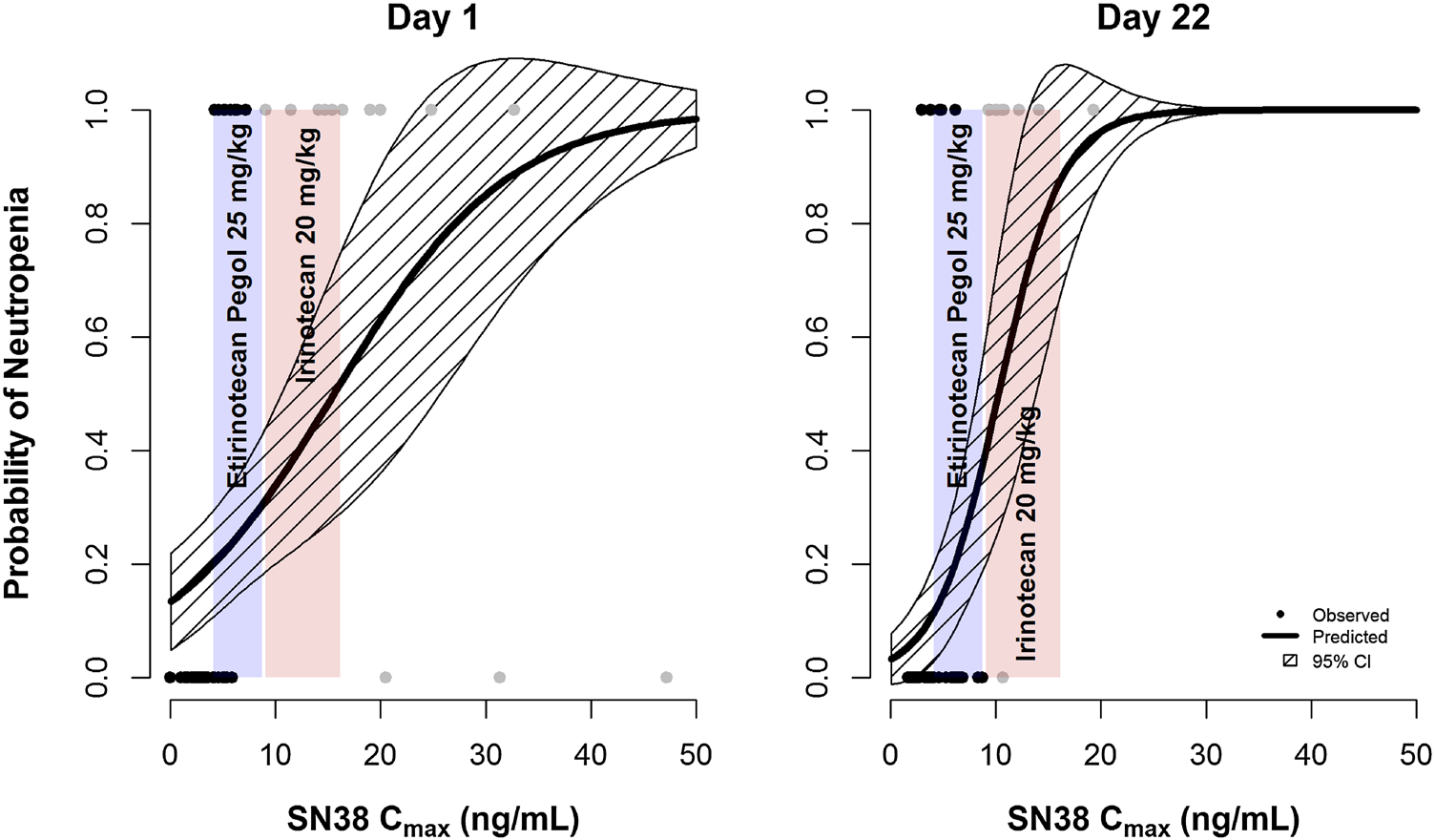
Logistic regression model showing the probability of neutropenia as a function of SN38 *C*_max_. *Black* and *gray points* represent *C*_max_ values after etirinotecan pegol and irinotecan once-weekly administration and whether neutropenia was observed (*p* = 1) or not (*p* = 0). The *blue-shaded area* represents SN38 *C*_max_ range after 25 mg/kg etirinotecan pegol and the *red-shaded area* represents SN38 *C*_max_ range after 20 mg/kg irinotecan, both administered once per week

Figure 4 shows that the probability of neutropenia increases with increasing SN38 *C*_max_ values. The blue-shaded area indicates the range of SN38 *C*_max_ values after 25 mg/kg EP, whereas the red-shaded area is the range of values after 20 mg/kg irinotecan. Irinotecan administration is associated with a higher risk of developing neutropenia, even at lower SN38 exposures than EP.

In contrast to SN38 *C*_max_, no relationship was found between SN38 AUC_0–168h_ and incidence of neutropenia (Fig. 3). The majority (71%) of irinotecan-treated dogs had SN38 AUC_0–168h_ values between 30 and <100 ng h/mL, while the majority of EP-treated dogs (96%) exhibited AUC_0–168h_ values ≥100 ng h/mL. Despite fivefold greater SN38 systemic exposure after EP compared to irinotecan, only 22% of EP-treated dogs developed neutropenia.

**Fig. 5:**
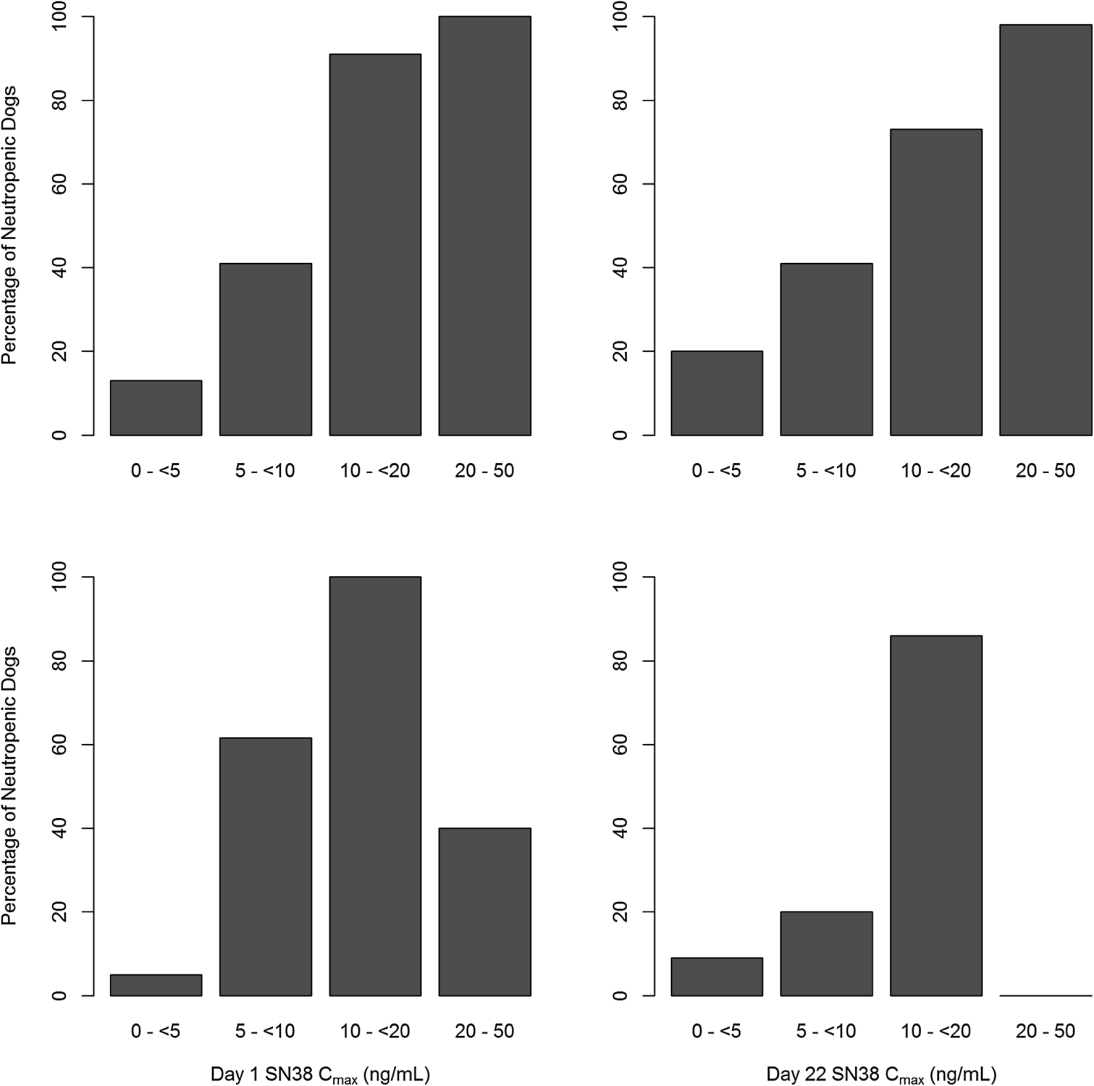
Visual predictive check comparing the predicted fraction of dogs with neutropenia from the logistic regression model (*top*) within each SN38 *C*_max_ bin with the actual fraction of dogs with neutropenia within the pre-specified bin range (*bottom*)

The logistic regression model was qualified by simulating 1000 uniformly distributed SN38 *C*_max_ values ranging from 0 to 50 ng/mL and estimating the incidence of neutropenia using the model parameters. Figure 5 shows that the model-predicted incidence of neutropenia as a function of SN38 *C*_max_ (top row) is similar to the observed incidence (bottom row), except for the 20–50 ng/mL *C*_max_ range, where many dogs in the 25 mg/kg irinotecan group died prior to reaching the neutrophil cutoff for neutropenia.

## Discussion

EP administration avoids high SN38 *C*_max_ and development of dose-limiting neutropenia observed after irinotecan, while maintaining reduced yet sustained plasma SN38 concentrations between doses. The probability of a neutropenia incidence was estimated to be between 0.1 and 0.4 for EP administered at an irinotecan-equivalent dose of 25 mg/kg once weekly compared to 0.3 to >0.9 with irinotecan administered at 20 mg/kg once weekly. Neutropenia occurred in approximately 80% of dogs receiving irinotecan (mean SN38 *C*_max_ of 13.5–26.3 ng/mL for 20 and 25 mg/kg). No neutropenia occurred in dogs receiving EP up to an irinotecan-equivalent dose of 25 mg/kg (mean SN38 *C*_max_ of 3.4–4.9 ng/mL for 20 and 25 mg/kg), even though SN38 AUC after EP was 2.5–3.6 times greater than that for irinotecan when both drugs were administered on the same schedule at doses delivering the same amount of irinotecan. EP is designed to achieve a reduced *C*_max_ but sustained exposure of SN38 to provide an enhanced anti-tumor activity and a better safety profile compared to irinotecan in animal models [12]. In a previous study on preclinical efficacy, EP outperformed irinotecan in tumor growth suppression and regression at equivalent or lower doses [12]. Animals treated with EP exhibited lasting tumor growth suppression and marked regression; complete regression continued for weeks after administration of the last EP dose in all tumor models. In contrast, animals treated with irinotecan at the maximum tolerated dose had limited and temporary tumor growth inhibition. EP also has better safety profile with significantly lesser severity of adverse events at an equivalent or lesser dose compared to irinotecan, as demonstrated in this study. The results from these two studies indicate that the extent or duration of exposure to SN38, characterized by AUC, likely determines its anti-tumor efficacy, whereas peak SN38 concentration determines the degree of toxicity. The results were consistent with a protracted infusion schedule of irinotecan in mice bearing xenograft of human tumors that showed more effective tumor regression [19].

The SN38 AUC after EP administration represents eightfold and fourfold increase in steady-state exposure compared to equivalent doses of irinotecan at 20 and 25 mg/kg once weekly, respectively. The slow release of irinotecan from EP is thought to not overwhelm the capacity of the carboxyl-esterase enzymes that deacetylate irinotecan to SN38 [20], thus allowing for a more efficient drug activation and resulting in greater SN38 AUC after EP administration than that of irinotecan administration. The exposure inside the tumor cells was previously shown to be increased by 300-fold compared with conventional irinotecan. The accumulation of SN38 in the tumor in the EP-treated animals was also sustained for a long duration [12], attributed to the extravasation through the leaky vasculature leading to an enhanced permeability and retention effect [21–23].

With EP resulting in an effective SN38 half-life of >40 days in humans [24], it can provide sustained SN38 exposure throughout the dosing interval. In cancer patients, after the fourth dose of 145 mg/m^2^ of EP administered every 21 days, corresponding SN38 *C*_max_ was approximately 5 ng/mL, cumulative AUC of approximately 2800 ng.h/mL, and trough concentrations of approximately 1 ng/mL [24]. In comparison, four cycles of 350 mg/m^2^ irinotecan administered every 21 days result in a SN38 *C*_max_ of 19 ng/mL, a cumulative AUC of 1300 ng h/mL, and concentrations of >1 ng/mL for less than 24 h post-each dose [25, 26]. Similar to the data presented in dogs, in the Phase 3 BEACON study, where 145 mg/m^2^ EP was administered every 21 days to patients with advanced or metastatic breast cancer, the rate of grade ≥3 neutropenia was 10% [27], while the Camptosar package insert mentions a 22% rate of grade ≥3 neutropenia in the patients [28].

In conclusion, EP avoids the high SN38 *C*_max_ and reduces the incidence of dose-limiting neutropenia that is frequently observed with irinotecan administration while maintaining sustained SN38 exposure. The incidence of neutropenia in dogs receiving weekly doses of irinotecan or EP is strongly dependent on the magnitude of plasma SN38 *C*_max_, but not SN38 AUC.

## Electronic supplementary material

Below is the link to the electronic supplementary material.

Supplementary material 1 (DOCX 312 kb)
