# Supplementary material for: Etirinotecan pegol administration is associated with lower incidences of neutropenia compared to irinotecan administration
Source: Cancer Chemother Pharmacol. 2016 Nov 30;79(1):57–67. doi: 10.1007/s00280-016-3192-6 (PMC5225190; doi:10.1007/s00280-016-3192-6)

**Model development and qualification of SN38 population pharmacokinetic model after etirinotecan pegol and irinotecan**

**Excluded Data from Analysis**

Two animals (4004D and 4504E from the 25 mg/kg etirinotecan pegol group) with approximately 3- to 4-fold higher SN38 concentrations for the same dose were excluded from the analysis because of their significantly higher SN38 concentrations. Neither dogs experienced neutropenia.

**SN38 pharmacokinetics using non-linear mixed effects modeling**

Population pharmacokinetic parameters determined from SN38 after irinotecan and etirinotecan pegol administration are listed in Tables S1 and S2, respectively. Goodness-of-fit plots are shown in Figures S1 and S2; the structural models adequately described observed SN38 concentrations after administration of irinotecan or etirinotecan pegol, respectively. There is good agreement between the individual predicted and observed concentrations without bias in the conditional weighted residuals over time or across predicted concentration values. Conditional weighted residuals were within the absolute 4 unit ordinates. Prediction- and variability-corrected visual predictive check captured majority of the observed data within the 95% prediction interval (Figure 3).

Population pharmacokinetic model-predicted AUC_0-48h_ values were compared to those obtained using non-compartmental analysis. As shown in Figure 4, there was good correlation between the two estimation approaches, indicating that the population pharmacokinetic models were suitable for predicting AUC_0-168h_ values corresponding to one dosing interval, for all animals across the two studies

Table S1: Population pharmacokinetic model parameters of SN38 after 1-hour intravenous infusion of irinotecan in dogs

| Parameter | Final model  Mean (SE) | Shrinkage (%) | Boostrap  Median [95% PI] |
| --- | --- | --- | --- |
| Structural model parameters |  |  |  |
| Apparent clearance (CL, mL/h/kg) | 2360 (361) |  | 2312 (1746, 3053) |
| Apparent volume of central compartment (V_c_, mL/kg) | 7760 (1070) |  | 7694 (6283, 8818) |
| Apparent volume of peripheral compartment (V_p_, mL/kg) | 18200 (4100) |  | 17824 (13971, 24370) |
| Intercompartmental clearance (Q, mL/h/kg) | 4030 (878) |  | 3948 (2752, 5279) |
| Interindividual variability |  |  |  |
| %CV of CL | 55.6 (32) | 1.3 | 54.4 (37.8, 71.3) |
| %CV of V_c_ | 23.7 (21.6) | 31.1 | 24.3 (4.64, 38.7) |
| %CV of Q | 52.2 (64.7) | 21.8 | 52.9 (11.8, 99.3) |
| Residual variability |  |  |  |
| Proportional residual error (%) | 26 (17.3) | 15.4 | 25 (17, 34) |

CV, coefficient of variation; SE, standard error; PI, prediction interval

Table S2: Population pharmacokinetic model parameters of SN38 after 1-hour intravenous infusion of etirinotecan pegol in dogs

| Parameter* | Final model  Mean (SE) | Shrinkage (%) |
| --- | --- | --- |
| Structural model parameters |  |  |
| Apparent clearance (CL, mL/h/kg) | 22.9 (2.63) |  |
| Apparent volume of central compartment (V_c_, mL/kg) | 6800 (463) |  |
| Apparent volume of peripheral compartment (V_p_, mL/kg) | 5170 (586) |  |
| Intercompartmental clearance (Q, mL/h/kg) | 93.5 (13.2) |  |
| Appearance of SN38 rate constant (K_in_, 1/h) | 3.63 (1.77) |  |
| Interindividual variability |  |  |
| %CV of CL | 72.9 (19.1) | 11.1 |
| %CV of V_c_ | 26.1 (3.17) | 12.5 |
| %CV of Q | 42.2 (19.4) | 65.2 |
| Interoccasion variability |  |  |
| %CV of CL | 49.4 (6.88) | 36.7 |
| %CV of V_c_ | 26.1 (3.67) | 32.1 |
| Corr(CL,V_c_) | 96.2 (6.35) |  |
| Residual variability |  |  |
| Proportional residual error (%) | 21.4 (0.49) | 15.3 |

CV, coefficient of variation; SE, standard error; PI, prediction interval

*Median and 95% prediction interval from bootstrap resampling not obtained due to very long run time for this model.

Figure S1: Goodness-of-fit plots for the SN38 population pharmacokinetic model after irinotecan administration


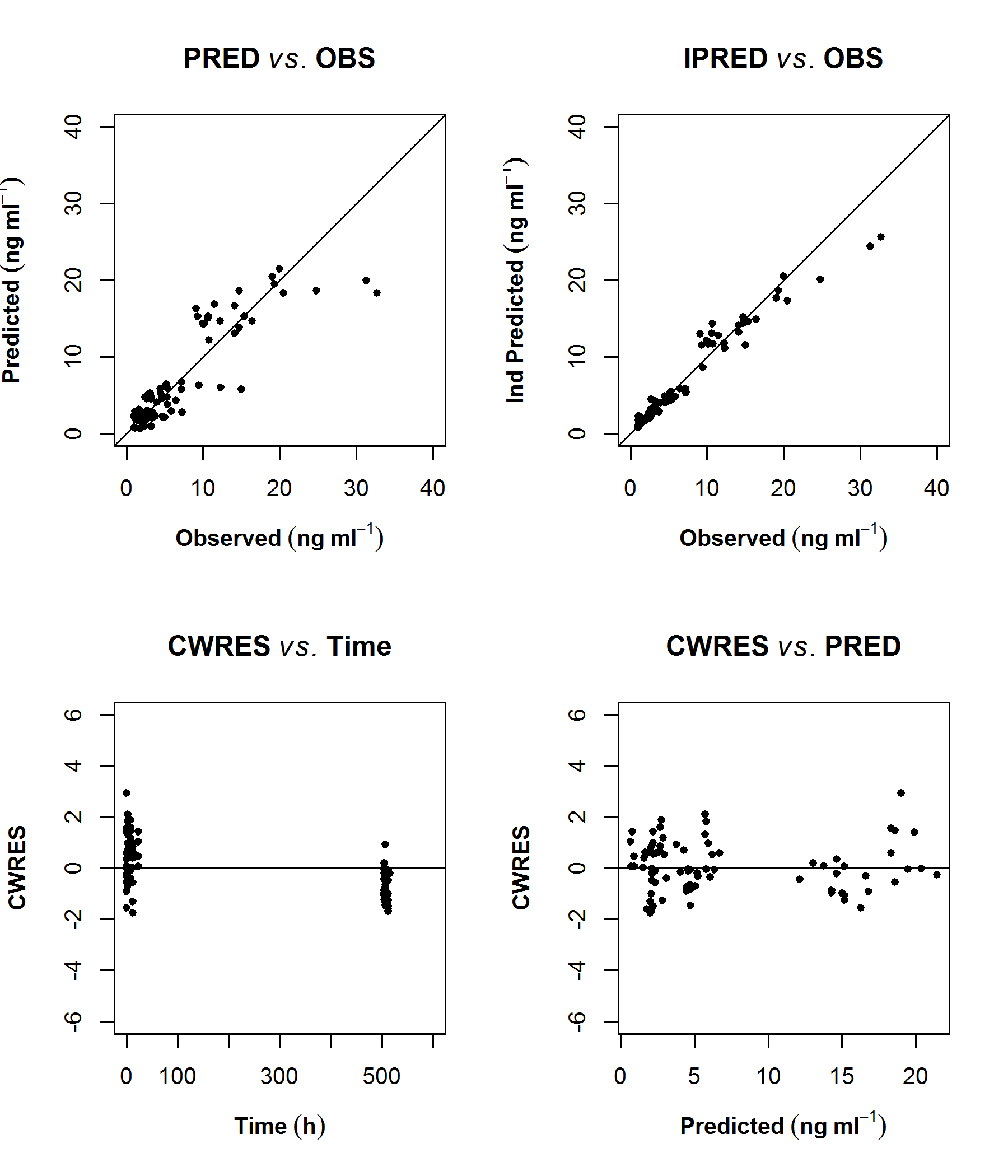


OBS: observed data; PRED: population-predicted data; IPRED: individual Bayesian predicted data; CWRES: conditional weighted residuals

Figure S2: Goodness of fit plots for the SN38 population pharmacokinetic model after etirinotecan pegol administration


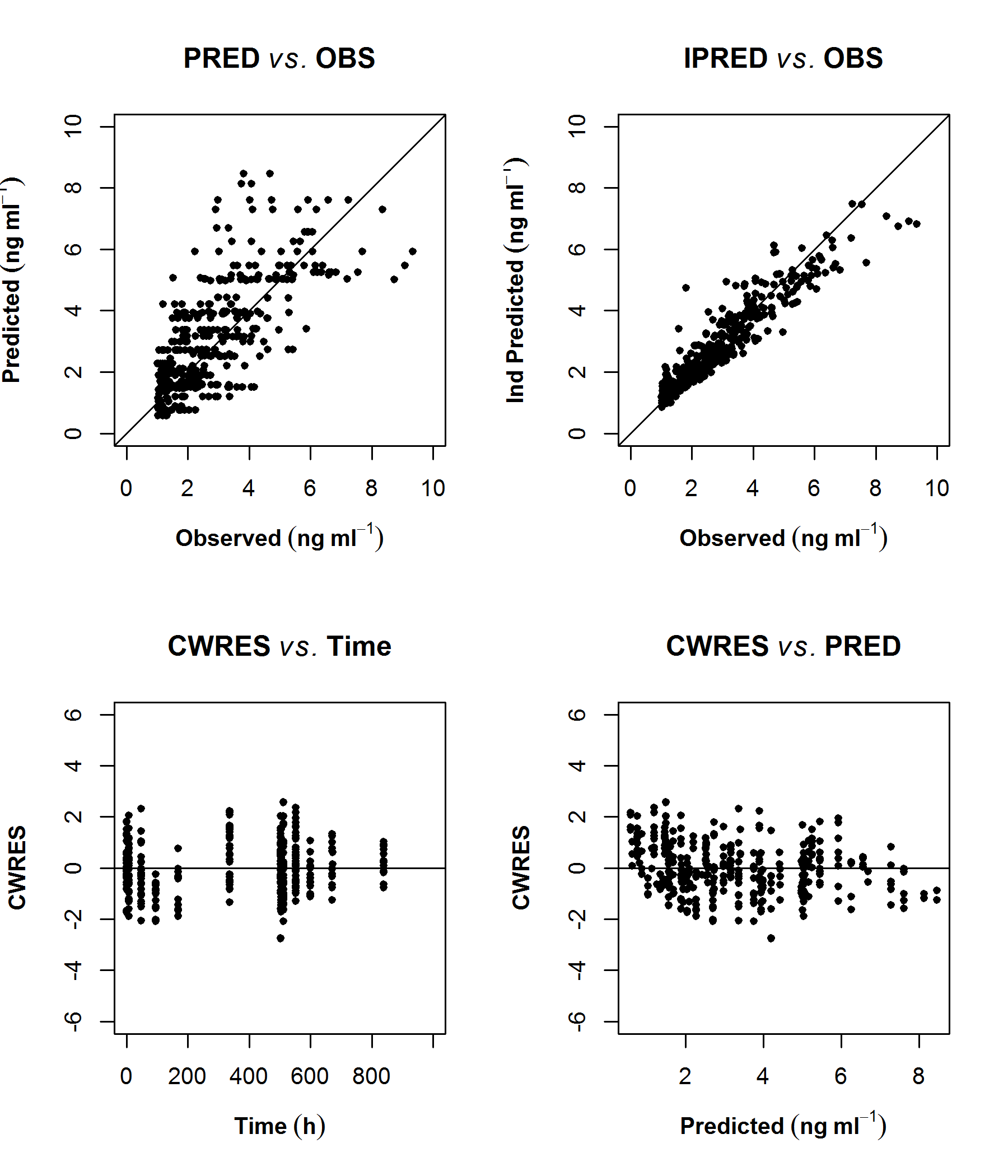


OBS: observed data; PRED: population-predicted data; IPRED: individual Bayesian predicted data; CWRES: conditional weighted residuals

Figure 3: Prediction- and variability-corrected visual predictive check for SN38 concentrations after irinotecan (top) and etirinotecan pegol (bottom) administration after Day 1 and Day 22 once-weekly doses


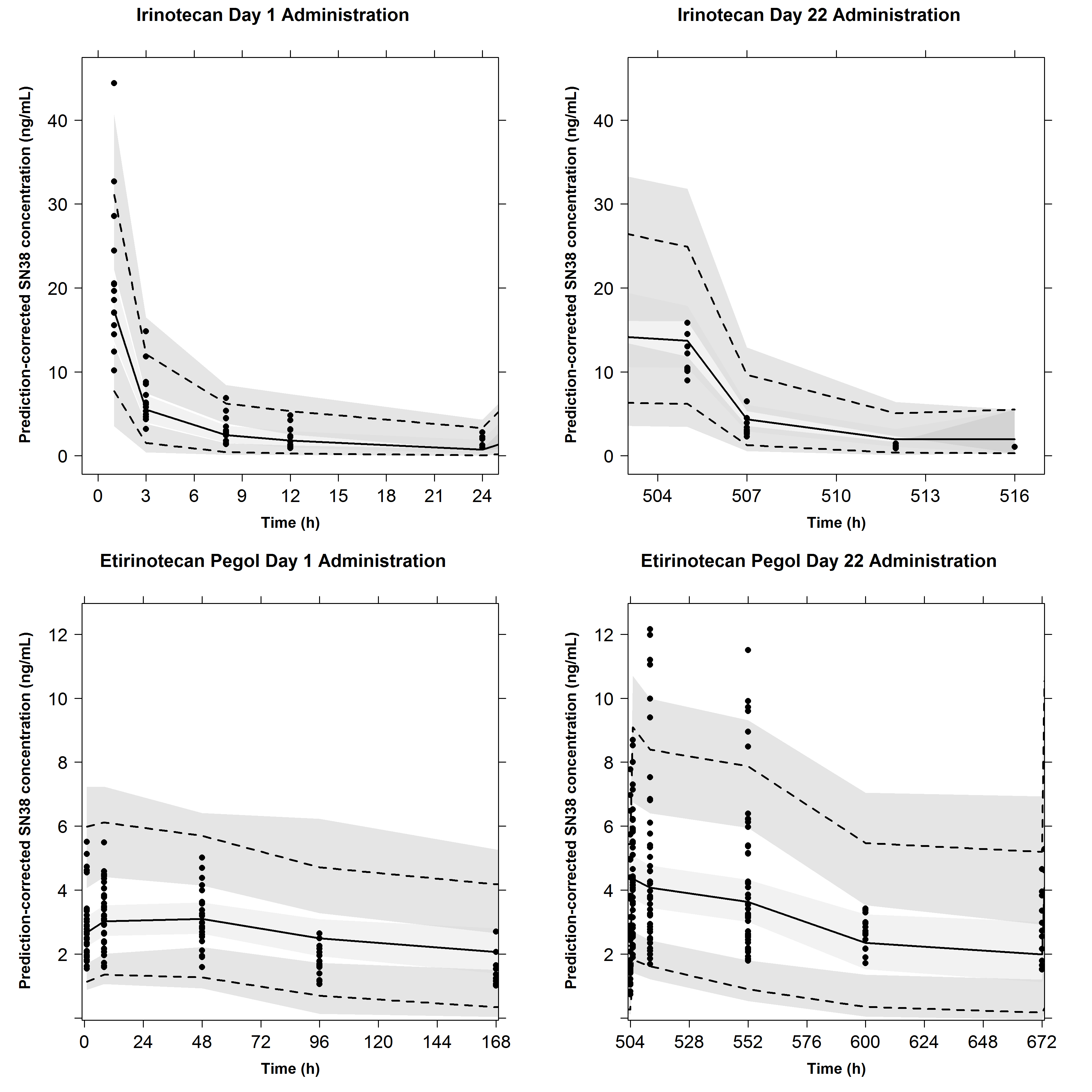


Figure 4: Correlation between SN38 AUC_0-48h_ values computed using non-compartmental analysis and population pharmacokinetic modeling


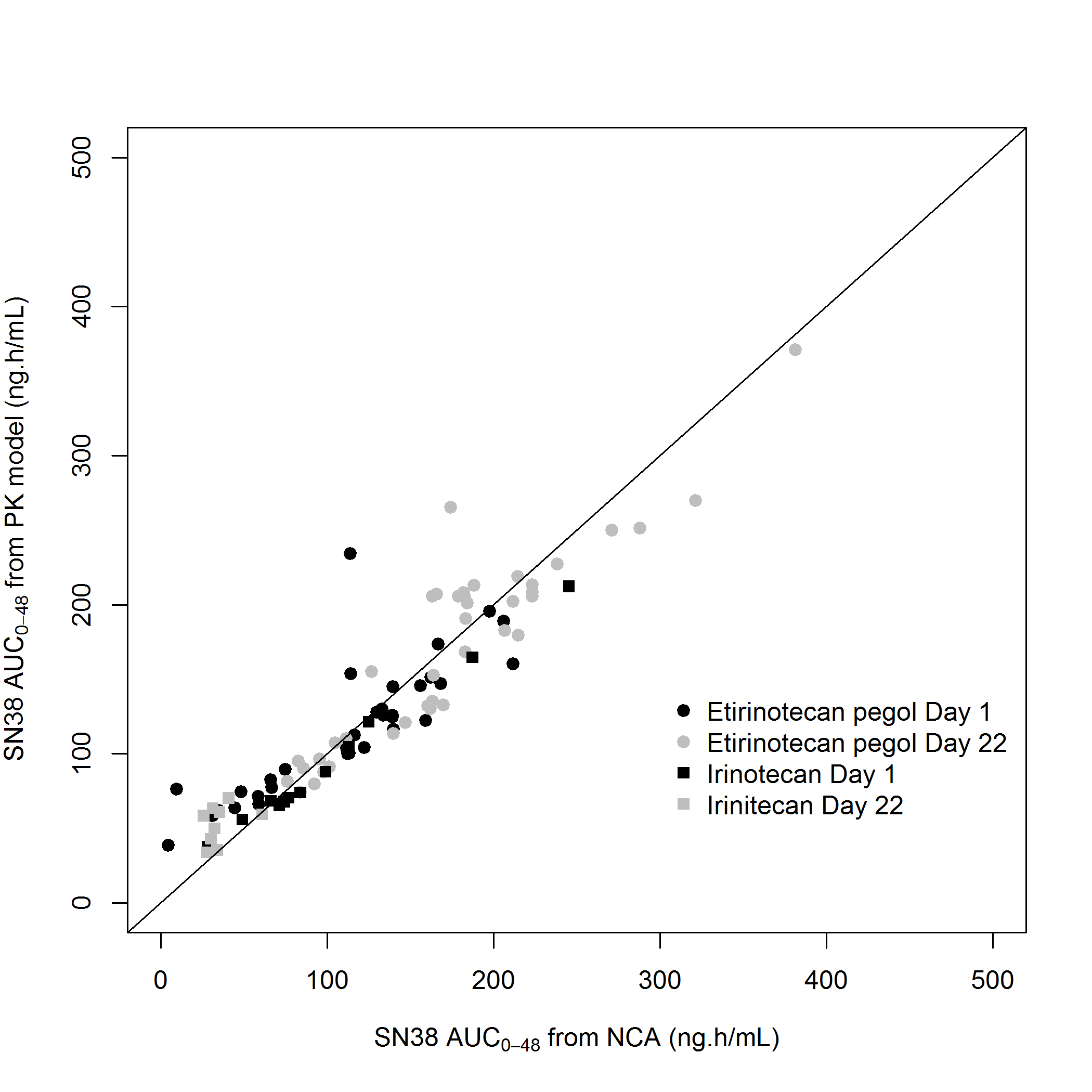

Supplement: Supplementary file 1 — Supplementary material 1 (DOCX 312 kb) [file 280_2016_3192_MOESM1_ESM.docx]
